# Triple Independent Segmental Drainage of the Brachial Veins Into the Basilic Vein Associated With Variant Formation of the Median Nerve: A Cadaveric Case Report

**DOI:** 10.7759/cureus.110589

**Published:** 2026-06-10

**Authors:** Angelos Kandilas, George Triantafyllou, Alexandros Samolis, Konstantinos Natsis, Maria Piagkou

**Affiliations:** 1 Department of Anatomy, School of Medicine, Faculty of Health Sciences, National and Kapodistrian University of Athens, Athens, GRC; 2 Department of Anatomy and Surgical Anatomy, School of Medicine, Faculty of Health Sciences, Aristotle University of Thessaloniki, Thessaloniki, GRC

**Keywords:** anatomical variation, axillary vein, basilic vein, brachial veins, median nerve

## Abstract

Variations of the upper-limb venous system are relatively common; however, complex segmental venous drainage patterns associated with neural variations remain exceptionally rare. Detailed knowledge of these anatomical configurations is clinically important because the basilic and brachial veins are frequently involved in vascular access procedures, venous catheterization, reconstructive microsurgery, and axillary surgery.

The present cadaveric case report describes a rare neurovascular variation identified during routine educational dissection of the left upper limb of a 75-year-old male donor. The variation consisted of triple independent segmental drainage of the brachial veins into the basilic vein, resulting in a non-classical multilevel formation of the axillary vein. The first brachial vein drained into the basilic vein at the middle third of the arm, the second near the origin of the ulnar nerve, and the third near the formation of the median nerve. Additionally, the median nerve demonstrated a variant formation consisting of two lateral roots and one medial root. Of particular anatomical and clinical significance, the third brachial vein coursed anterior to the medial root of the median nerve before draining into the basilic vein.

The coexistence of multiple venous and neural variations resulted in a complex neurovascular arrangement in the axillary region. Such anatomy may increase the risk of hemorrhagic or neural complications during venous catheterization, brachiobasilic arteriovenous fistula creation, axillary lymph node dissection, brachial plexus exploration, and reconstructive vascular procedures. Particular caution may be warranted during ultrasound-guided venous cannulation, peripherally inserted central catheter placement, and brachiobasilic fistula transposition. The present case highlights the importance of recognizing rare combined neurovascular variations and performing careful preoperative vascular mapping to minimize complications during upper-limb surgical and interventional procedures.

## Introduction

The venous anatomy of the upper limb demonstrates considerable variability, particularly regarding the formation and drainage patterns of the brachial, basilic, and axillary veins. In the classical anatomical arrangement, the paired brachial venae comitantes accompany the brachial artery and unite proximally before draining into the basilic vein near the inferior border of the teres major muscle to form the axillary vein [1]. Although variations involving duplication, asymmetry, accessory venous channels, and atypical superficial-deep venous communications have been documented, most are clinically silent and discovered incidentally during surgical or radiological procedures [2,3]. Recent anatomical reviews continue to demonstrate substantial variability in brachial and basilic venous anatomy, including alterations in venous confluence patterns and drainage pathways [4].

Detailed knowledge of upper-limb venous anatomy has become increasingly important because the basilic and brachial veins are frequently involved in vascular access procedures, venous catheterization, reconstructive microsurgery, and the creation of brachiobasilic arteriovenous fistulae for hemodialysis [5,6]. Anatomical variations affecting venous drainage patterns may complicate surgical dissection, alter venous outflow, and increase the risk of thrombosis, hemorrhage, catheter malposition, or fistula failure [7,8]. Furthermore, the close anatomical relationship between the venous system and the brachial plexus makes these variations particularly relevant during axillary surgery and neurovascular exploration. Failure to recognize such variants during ultrasonographic or venographic evaluation may also lead to inaccurate vascular mapping and procedural complications.

Variability of the brachial plexus itself is well recognized and includes alterations in cord formation, branching patterns, and root communications [9,10]. However, the coexistence of multiple venous anomalies with variant neural configurations within the same anatomical region is rarely described. Such combined neurovascular variations may create a crowded surgical field and increase the risk of iatrogenic injury during invasive procedures. Recent reports describing a fenestrated basilic vein traversed by a variant medial brachial cutaneous nerve and a subclavian vein fenestration traversed by a brachial plexus branch have further highlighted the developmental and clinical significance of combined neurovascular variations in the upper limb [11,12]. To the authors’ knowledge, the coexistence of triple independent segmental brachial venous drainage, variant formation of the median nerve, and intimate neurovascular relationships is highly unusual.

Development of the upper-limb venous system involves the formation and remodeling of a primitive venous plexus, with selective regression and persistence of embryonic channels. Variations of the brachial, basilic, and axillary veins may result from incomplete regression or altered remodeling during this process. Because neural and vascular structures develop concurrently within the developing limb bud, combined neurovascular variations may occasionally occur and may reflect shared developmental mechanisms [12,13].

The present report describes a rare case of triple independent segmental drainage of the brachial veins into the basilic vein, resulting in a non-classical multilevel formation of the axillary vein, accompanied by variant formation of the median nerve and intimate neurovascular relationships within the axillary region. The anatomical configuration, embryological considerations, radiological relevance, and potential clinical implications of this unusual variation are discussed.

## Case presentation

During routine educational dissection at the Department of Anatomy and Surgical Anatomy, an unusual venous configuration was identified in the left upper limb of a 75-year-old embalmed male cadaver. The body had been donated to the institutional body donation program after the donor provided written informed consent prior to death, in accordance with institutional ethical standards for anatomical education and research. The variation involved the brachial venae comitantes and their contribution to the formation of the axillary vein.

In the typical anatomical pattern, the paired brachial veins accompany the brachial artery and unite proximally before joining the basilic vein near the inferior border of the teres major muscle to form the axillary vein. In the present specimen, however, three independent brachial venous trunks drained separately into the basilic vein at different anatomical levels, resulting in a segmental and multilevel formation of the axillary vein. The first brachial vein joined the basilic vein approximately at the middle third of the arm. A second venous confluence was identified more proximally, near the level where the ulnar nerve arose from the medial cord of the brachial plexus. The third brachial vein drained into the basilic vein near the formation of the median nerve and coursed anterior to its medial root before terminating. This relationship created a particularly close association between the anomalous venous channel and the neural structures of the axillary region. Based on its course and relationship to the brachial artery, the third venous trunk appeared to represent a persistent brachial vena comitans that drained independently into the basilic vein rather than uniting with its companion vein.

**Figure 1:**
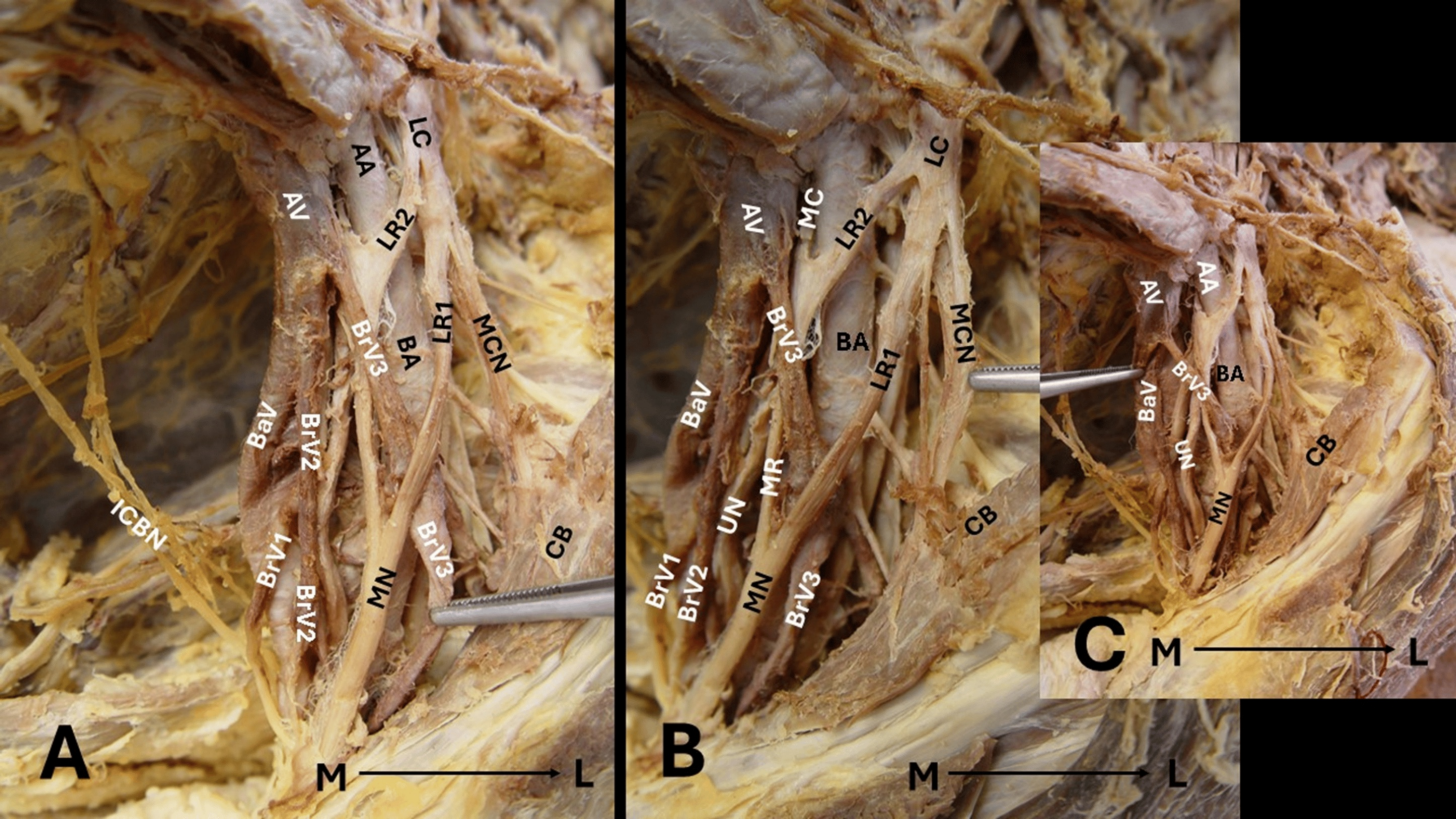
Dissection of the left axillary region and proximal arm demonstrating an unusual multilevel formation of the axillary vein (AV) through triple independent drainage of the brachial veins (BrVs) into the basilic vein (BaV). (A) The first brachial vein (BrV1) drains into the basilic vein at the middle third of the arm. (B) The second brachial vein (BrV2) joins the basilic vein more proximally, near the origin of the ulnar nerve (UN). (C) The third brachial vein (BrV3) courses anterior to the medial root (MR) of the median nerve (MN) before draining into the basilic vein. Variant formation of the median nerve by two lateral roots (LR1, LR2) arising from the lateral cord (LC) and one medial root originating from the medial cord (MC) of the brachial plexus is also demonstrated. The close anatomical relationships between the anomalous venous channels and the cords and terminal branches of the brachial plexus create a crowded neurovascular arrangement within the axillary region and proximal arm. CB: Coracobrachialis muscle. Orientation: M: Medial; L: Lateral.

An additional anatomical variation involved the formation of the median nerve, which was formed by two lateral roots arising from the lateral cord and one medial root originating from the medial cord of the brachial plexus. The third brachial vein crossed anterior to the medial root of the median nerve immediately before draining into the basilic vein. Furthermore, the second venous confluence was located near the origin of the ulnar nerve, while all venous channels were situated in close proximity to the cords and terminal branches of the brachial plexus, producing a crowded neurovascular arrangement within the axilla and proximal arm (Figure 1).

No evidence of previous trauma, surgical intervention, or pathological alteration was identified in the dissected region. The surrounding arterial anatomy demonstrated no major variation. The findings were documented through meticulous anatomical dissection and photographic recording.

According to the classification proposed by Lee HS et al. [14], the present configuration corresponded to a Type A1 pattern of axillary vein formation; however, the presence of three independent venous terminations represented a particularly extensive and discontinuous segmental arrangement. To our knowledge, this degree of segmental brachial venous drainage associated with variant formation of the median nerve has not been previously reported. The observed anatomy may have important implications for axillary surgery, venous catheterization, vascular reconstruction, brachial plexus exploration, ultrasonographic vascular mapping, and brachiobasilic arteriovenous fistula creation for hemodialysis access, particularly given the intimate relationship between the anomalous venous channels and neighboring neural structures.

## Discussion

The present case describes a rare variation of the upper-limb venous system characterized by triple independent drainage of the brachial veins into the basilic vein, resulting in a non-classical multilevel formation of the axillary vein. This configuration corresponds to a Type A1 variation, in which venous drainage occurs through multiple segmental confluences rather than through a single proximal union of the paired brachial veins [14]. In the typical anatomical pattern, two venae comitantes accompany the brachial artery and unite proximally before draining into the basilic vein near the inferior border of the teres major muscle [1,3]. In the present specimen, however, three distinct brachial venous trunks drained separately into the basilic vein at different anatomical levels, producing a discontinuous and staggered pattern of venous drainage. Although upper-limb venous variations are relatively common, most reported cases involve duplication, asymmetry, accessory venous channels, or altered superficial-deep venous communications, whereas triple independent segmental drainage of the brachial veins into the basilic vein appears to be exceptionally rare [2,4].

An additional noteworthy feature of the present case was the coexistence of variant neural anatomy within the axillary region. The median nerve was formed by two lateral roots and one medial root, while the third brachial vein coursed anterior to the medial root of the median nerve before draining into the basilic vein. Furthermore, the second venous confluence was identified near the origin of the ulnar nerve. These findings demonstrate a close topographical relationship between the anomalous venous channels and the components of the brachial plexus. Variability in brachial plexus formation, including differences in cord organization, branching patterns, and neural interconnections, has been well documented [9,10]. However, the coexistence of multiple venous and neural variations within the same confined anatomical region creates a particularly complex neurovascular environment that may increase the risk of surgical complications.

The clinical significance of this arrangement is considerable. The basilic and brachial veins are commonly used in vascular access procedures, particularly in the creation of brachiobasilic arteriovenous fistulas for hemodialysis [5,6]. Previous studies have emphasized that unexpected venous tributaries and anatomical variability may complicate basilic vein transposition, increase operative difficulty, and predispose patients to thrombosis, hemorrhage, or fistula failure [8]. In the present case, multiple independent venous drainage sites may complicate venous mobilization, superficialization, and surgical orientation during axillary or vascular procedures. Moreover, the intimate relationship between the anomalous veins and adjacent neural structures may increase the risk of iatrogenic nerve injury during axillary lymph node dissection, venous catheterization, brachial plexus exploration, and reconstructive surgery. Similar concerns have been raised in studies investigating the anatomical relationship between veins and neighboring nerves during venous access and fistula elevation procedures [7,15]. Particular caution may be warranted during ultrasound-guided venous cannulation, peripherally inserted central catheter placement, brachiobasilic fistula transposition, and axillary surgical procedures. Failure to recognize such variations during ultrasonographic or venographic evaluation may also result in inaccurate vascular mapping and procedural complications.

Embryologically, the observed variation may reflect the persistence of multiple primitive venous channels from the embryonic upper-limb venous plexus, accompanied by altered remodeling of adjacent neural structures during brachial plexus development. Incomplete regression of embryonic venous channels is a recognized mechanism underlying unusual venous drainage patterns and may explain the persistence of the multiple segmental brachial venous trunks observed in the present specimen [13]. Recent reports have further highlighted the developmental relationship between neural and venous structures in the upper limb. Triantafyllou G et al. described a fenestrated basilic vein traversed by a variant medial brachial cutaneous nerve [12], while Silawal S et al. reported a subclavian vein fenestration traversed by a brachial plexus branch [11]. These findings support the concept that neural elements and the primitive venous plexus develop in close spatial and temporal association and that variations affecting one system may coexist with variations affecting the other. Although the developmental interpretation proposed in the present case remains speculative because no embryological or histological confirmation was available, the coexistence of venous and neural variations may reflect concurrent alterations in neurovascular morphogenesis during upper-limb development. Therefore, the present case not only represents a rare anatomical finding but also demonstrates a potentially hazardous surgical configuration that may complicate vascular access procedures, brachiobasilic fistula creation, axillary surgery, and brachial plexus exploration.

The present report is limited by its cadaveric nature and the examination of a single specimen, which precludes assessment of the prevalence or variability of this anatomical configuration in the general population. Additionally, no radiological correlation or intraoperative confirmation was available; therefore, the functional hemodynamic implications of the variation could not be evaluated. Although the surrounding neurovascular structures were carefully dissected, histological or embryological confirmation of the developmental mechanisms underlying the observed venous and neural variations was not possible. Nevertheless, detailed anatomical documentation of this rare configuration may improve recognition of clinically significant upper-limb neurovascular variations.

## Conclusions

The present case describes a rare anatomical variation characterized by triple independent segmental drainage of the brachial veins into the basilic vein, resulting in a non-classical multilevel formation of the axillary vein. The coexistence of variant formation of the median nerve and intimate neurovascular relationships within the axillary region created a particularly complex anatomical arrangement with important clinical implications. The atypical topography of the venous channels, especially the vein coursing anterior to the medial root of the median nerve, may increase the risk of iatrogenic injury during vascular access procedures, axillary surgery, venous catheterization, brachial plexus exploration, and brachiobasilic arteriovenous fistula creation. Particular caution may be warranted during ultrasound-guided venous cannulation, peripherally inserted central catheter placement, brachiobasilic fistula transposition, axillary lymph node dissection, and brachial plexus surgery. Recognition of such combined neurovascular variants is essential for accurate preoperative vascular mapping and for minimizing complications during surgical and interventional procedures involving the upper limb and axillary region. Awareness of these variations is important for surgeons, radiologists, and interventionalists operating within the axillary region.
